# Prevalence of mental disorders, psychosocial distress and need for psychosocial support in cancer patients – study protocol of an epidemiological multi-center study

**DOI:** 10.1186/1471-244X-12-70

**Published:** 2012-07-02

**Authors:** Anja Mehnert, Uwe Koch, Holger Schulz, Karl Wegscheider, Joachim Weis, Hermann Faller, Monika Keller, Elmar Brähler, Martin Härter

**Affiliations:** 1 Department and Outpatient Clinic of Medical Psychology, University Medical Center Hamburg-Eppendorf, Martinistrasse 52, 20246, Hamburg, Germany; 2 Department of Medical Biometry and Epidemiology, University Medical Center Hamburg-Eppendorf, Martinistr. 52, 20246, Hamburg, Germany; 3 Department of Psychooncology, Tumor Biology Center, University of Freiburg, Breisacher Str. 117, 79106, Freiburg, Germany; 4 Institute of Psychotherapy and Medical Psychology, University of Würzburg, Klinikstr. 3, 97070, Würzburg, Germany; 5 Division of Psychooncology, Department for Psychosomatic and General Clinical Medicine, University Hospital Heidelberg, Im Neuenheimer Feld 155, 69120, Heidelberg, Germany; 6 Department of Medical Psychology and Medical Sociology, University Medical Center Leipzig, Philipp-Rosenthal-Str. 55, 04103, Leipzig, Germany

## Abstract

**Background:**

Empirical studies investigating the prevalence of mental disorders and psychological distress in cancer patients have gained increasing importance during recent years, particularly with the objective to develop and implement psychosocial interventions within the cancer care system. Primary purpose of this epidemiological cross-sectional multi-center study is to detect the 4-week-, 12-month-, and lifetime prevalence rates of comorbid mental disorders and to further assess psychological distress and psychosocial support needs in cancer patients across all major tumor entities within the in- and outpatient oncological health care and rehabilitation settings in Germany.

**Methods/Design:**

In this multicenter, epidemiological cross-sectional study, cancer patients across all major tumor entities will be enrolled from acute care hospitals, outpatient cancer care facilities, and rehabilitation centers in five major study centers in Germany: Freiburg, Hamburg, Heidelberg, Leipzig and Würzburg. A proportional stratified random sample based on the nationwide incidence of all cancer diagnoses in Germany is used. Patients are consecutively recruited in all centers. On the basis of a depression screener (PHQ-9) 50% of the participants that score below the cutoff point of 9 and all patients scoring above are assessed using the Composite International Diagnostic Interview for Oncology (CIDI-O). In addition, all patients complete validated questionnaires measuring emotional distress, information and psychosocial support needs as well as quality of life.

**Discussion:**

Epidemiological data on the prevalence of mental disorders and distress provide detailed and valid information for the estimation of the demands for the type and extent of psychosocial support interventions. The data will provide information about specific demographic, functional, cancer- and treatment-related risk factors for mental comorbidity and psychosocial distress, specific supportive care needs and use of psychosocial support offers.

## Background

Cancer is one of the leading causes of morbidity and mortality worldwide [1,2]. An estimated 3.2 million new cancer cases were diagnosed in Europe in 2008 [1]. The most frequent forms of cancer were colorectal cancers (13.6% of all cancer cases), breast cancer (13.1%), lung cancer (12.2%) and prostate cancer (11.9%). In Germany, recent epidemiological estimates indicate a projected annual incidence rate for 2010 of about 450,000 cancer cases [3]. Cancer incidence statistics show an enhanced 5-year survival rate up to 62% for women and 57% for men, including all cancer stages. The average age of onset of cancer is 69 years for both genders. For the population of 82 million people in Germany, these developments lead to a prevalence of 1.4 million cancer patients within the range of five years post diagnosis and 2.1 million cancer patients in whom the diagnosis is back up to 10 years [3].

Given both the high cancer incidence and continuous advances in cancer detection, multimodal treatments and targeted therapies, the proportion of cancer survivors continues to grow in industrialized countries. Cancer survivorship covers a variety of medical conditions and periods that are divided into acute survival, middle and long-term survival including disease-free survival as well as cancer recurrence and chronic disease [4]. Thus, short, middle and long-term survivorship has significant implications for both clinical and psychosocial research as well as for health care services research [5-7].

Comprehensive patient-centered care in oncology has been emphasized in international guidelines and standards [8], implying cancer prevention and early detection as well as high quality evidence-based medical treatment, rehabilitation and palliative care. Improving the quality of care requires recognition and addressing patients’ distress, mental disorders and supportive care needs during treatment and after care. Thus, psychosocial and psycho-oncological support services considerably contribute to improving the quality of life of patients as a central outcome criterion of oncological care [9].

Emotional distress is common after a cancer diagnosis [10], and is often a result of a variety of problems that can affect every aspect of life according to different stages of the disease. Typical issues relate to physical symptoms and problems such as pain, functional impairments and states of chronic fatigue, family burden such as the uncertainty about individual roles and responsibilities, existential concerns such as isolation or meaning in one's life as well as social, financial and occupational problems [11].

Many cancer patients suffer from a high symptom burden, which can remain for months and years after the initial cancer therapy or can occur again in the face of long-term therapy or complications in the disease trajectory. High symptom burden is associated with a significant increase in feelings of helplessness and hopelessness and may adversely impact a patient’s quality of life [12-14]. The spectrum of emotional reactions and psychosocial consequences ranges along a continuum including anxiety, fear, sadness and depression, helplessness and hopelessness as well as adjustment disorders, anxiety disorders, posttraumatic stress disorder, depression, family conflicts or existential crises [11,15-17].

Empirical studies investigating the prevalence of mental disorders and the frequency of psychosocial burden in cancer patients have gained increasing importance during recent years [18]. This research is of particular significance for the development and implementation of psychosocial support offers within the health care system. Recent meta-analytical evidence indicates that the overall prevalence of mental disorders among cancer patients ranges from 9.8% to 38.2% in various cancer settings [19,20]. In an international review conducted by Mitchell et al. [19] including 94 interview-based studies, the prevalence of depression by DSM or ICD criteria in oncological and hematological settings (70 studies) was 16.3% (95% CI 13.4–19.5); the prevalence of dysthymia was 2.7% (95% CI 1.7–4.0); the prevalence of adjustment disorder was 19.4% (95% CI 114.5–24.8); and the prevalence of anxiety disorders was 10.3% (95% CI 5.1–17.0). However, combination diagnoses were prevalent among up to 38.2% (95% CI 28.4–48.6) of patients. The prevalence of depression by DSM or ICD criteria in palliative-care settings (24 studies) was 16.5% (95% CI 13.1–20.3); the prevalence of adjustment disorder was 15.4% (95% CI 10.1–21.6); the prevalence of anxiety disorders was 9.8% (95% CI 6.8–13.2); and combination diagnoses were prevalent among up 29.0% (95% CI 10.1–52.9) of patients. Accordingly, Singer et al. [20] observed prevalence rates up to 32% (95% CI 27–37) among cancer patients in acute hospitals.

However, few trials have examined the prevalence of mental disorders in cancer patients taking into account the wide spectrum of mental disorders including substance abuse or somatoform disorders, different health care settings as well as different tumor entities and disease stages for both genders. Fewer studies have examined the 4-week-, 12-month-, and lifetime prevalence rates of comorbid mental disorders in cancer patients [21] and the association between comorbid mental disorders and psychological distress.

The presence of a mental disorder or psychological symptom burden is not necessarily associated with subjective needs of patients for professional psychosocial support and the utilization of relevant offers [22]. Although a variety of psychological interventions have been shown to be effective in the reduction of psychosocial symptom distress and the improvement of quality of life [23-25], however, improving distress screening and the access of cancer patients to adequate psychosocial care remains a critical concern [26]. Thus epidemiological data about mental comorbidity, psychological symptom burden and supportive care needs are essential for the evidence based implementation of psychosocial support offers within oncological health care.

### Objectives

Although there has been extensive research looking at emotional distress among various cancer entities, there is limited evidence regarding prevalence rates of comorbid mental disorders in cancer patients across different tumor entities and care settings from an epidemiological point of view. Main research aim of this epidemiological cross-sectional multi-center study is to detect the 4-week-, 12-month-, and lifetime prevalence rates of comorbid mental disorders according to the ICD-10/DSM-IV (organic, including symptomatic, mental disorders, mental and behavioral disorders due to psychoactive substance use, mood disorders, neurotic, stress-related and somatoform disorders, and behavioral syndromes associated with physiological disturbances and physical factors); and to further assess psychological distress and psychosocial supportive care needs in cancer patients across all major tumor entities within the in- and outpatient oncological health care and rehabilitation settings in Germany.

Secondary aims are to examine the impact of demographic, functional, cancer- and treatment-related risk factors on the occurrence of mental comorbidity, psychosocial distress, specific supportive care needs and use of psychosocial support offers. Associations between mental comorbidity, psychosocial distress, supportive care needs, quality of life and medial decision-making will be also examined.

## Methods/Design

### Study design

**Figure 1:**
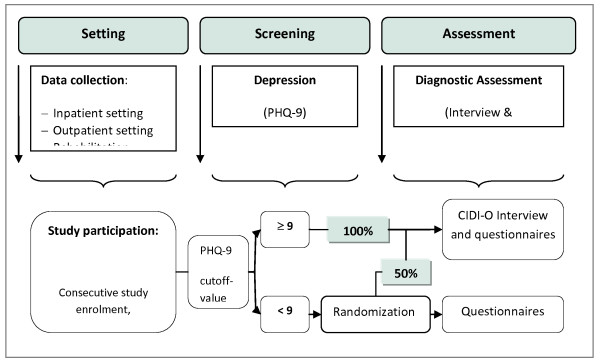
Overview over the research design.

In this multicenter, epidemiological cross-sectional study, cancer patients will be enrolled from acute care hospitals, outpatient cancer care facilities, cancer rehabilitation centers and clinics in five major study centers in Germany (Freiburg, Hamburg, Heidelberg, Leipzig and Würzburg). The centers are selected to represent various typical regions in Germany. Factors considered are the geographic location including new and old federal states, cities and towns, and different cancer care facilities. In each study center and surrounding areas, the university medical center, another hospital offering maximum medical care, three to four outpatient care facilities providing basic medical care and those serving as regional cancer centres as well as a minimum of two rehabilitation clinics are included for data collection. Patients are consecutively recruited in all centers. Figure 1 gives an overview over the research design.

### Study participants

Patient inclusion criteria contain the evidence of a malignant tumor and age between 18 through 75 years due to the validity of the Composite International Diagnostic Interview (M-CIDI) [27] which was adapted for cancer patients (CIDI-O). The oncology-specific adaption of the CIDI-O enhances the diagnostic spectrum of the M-CIDI by adding the diagnostic group of stress-related mental disorders, particularly due to cancer disease. A prominent modification strategy that fostered the development of the CIDI-O is the adding-on of cancer-related questions in the interview sections for depressive disorders, anxiety disorders and PTSD assessing different types of stressors and specifying time association of mental symptoms and stressors. Patients across all tumor entities and disease stages are included stratified by nationwide incidence of cancer diagnoses (see sample stratification). Patient exclusion criteria contain the presence of severe physical, cognitive and/or verbal impairments that would interfere with a patient’s ability to give informed consent for research.

### Recruitment and data collection

The study received research ethics committee approval in all involved federal states (The study was approved by the following Medical Associations: Hamburg: Ref. Nr. 2768, Schleswig-Holstein: Ref.-Nr. 61/09; Freiburg: Ref.-Nr. 244/07, Heidelberg: Ref.-Nr. S-228/2007; 50155039; Würzburg: Ref. Nr. 107/07; Leipzig: Ref. Nr. 200–2007).

All patients who fulfill the study inclusion criteria are contacted by study staff and consecutively recruited at the oncological health care settings and institutions in all centers. All patients provide written informed consent prior to participation. Principles of good research practice are strictly adhered to in this project including data and patient’s privacy protection.

### Interviewer training and quality standards

The study center Hamburg is responsible for the coordination of the data collection also ensuring the accuracy of the assessment and the data input. Study interviewers were trained extensively in a 1-2-day workshop on the use of the computer-based CIDI-O. After training, each interviewer conducted several test interviews of which one interview was videotaped and evaluated by one study center responsible for the quality assurance (Freiburg). Evaluation criteria included correct implementation of the interview questions, correctness of the patient information as well as interactional factors. Each interviewer received a detailed feedback on the implementation of the interviews. In addition, all conducted patient interviews were evaluated by the study center Freiburg. Incorrect interviews were excluded from the record.

### Randomization

All patients that score at the cutoff point of 9 or above at the PHQ-9 (total score) [28] are interviewed (CIDI-O), and 50% of the participants that score below this cutoff point are randomized to CIDI-O interviews. Block randomization is used in each center by each interviewer to allocate participants to CIDI-O interviews. Block size is defined by 20. Randomization is managed by using a computer-based randomization program. At the moment of randomization, the study interviewer enters the patient code and PHQ score into the program interface to obtain the assignment.

### Study measures

Table 1 gives an overview over the study measures.

**Table 1:** Overview over study measures

	**Participants**	
**Screening measure:** Patient Health Questionnaire (PHQ-9)	**PHQ-9: < 9**	**PHQ-9: ≥ 9**
**Standardized psychiatric interview**		
Composite International Diagnostic Interview for Oncology (CIDI-O)	x (50 %)	x
**Demographics, medical history and functional performance**		
Demographic characteristics	x	x
Medical characteristics	x	x
Karnofsky performance status scale	x	x
ECOG Scale	x	x
Brief Pain Inventory (BPI)	x	x
**Emotional distress and quality of life**		
General Anxiety Disorder-Scale (GAD-7)	x	x
Hospital Anxiety and Depression Scale – German version (HADS)	x	x
NCCN Distress Thermometer	x	x
EORTC Quality of Life Questionnaire (EORTC QLQ – C30)	x	x
**Psychosocial support and patient participation**		
Illness-specific Social Support Scale Short Version-8 (ISSS-8)	x	x
The Shared Decision Making Questionnaire (SDM-Q-9)	x	x
Control preference scale (CPS)	x	x
Information and supportive care needs and use of psychosocial support	x	x

#### Demographics, medical history and functional performance

Sociodemographic information is collected through use of a standardized self-report questionnaire (age, gender, marital status and partnership, children, school education, vocational training, monthly household income, employment status and occupational situation).

Medical information regarding tumor entity, date of and time since first and recurrent diagnosis, UICC disease stage, information about remission, cancer recurrence and progress, metastasis, curative or palliative treatment intention, past and current cancer treatments received as well as comorbid disorders is gathered through medical records.

The Karnofsky performance status scale [29] is a widely used performance measure for rating the ability of a somatically ill person to perform usual activities, evaluating a patient's progress after a therapeutic procedure, and determining a patient's suitability for therapy. The lower the Karnofsky score, the worse the survival for most serious illnesses such as cancer. A person is evaluated on a score ranging from 0 to 100, where 0 is “dead” and 100 is “normal, no complaints, and no signs of disease”.

The ECOG Scale [30,31] is used in addition to assess how a patient's disease is progressing, and to assess how the disease affects the daily living abilities of the patient. A person is evaluated on a score ranging from 0 to 5, where 0 is “fully active, able to carry on all pre-disease performance without restriction” and 5 is “dead”.

#### Composite international diagnostic interview for oncology (CIDI-O)

The M-CIDI was developed by the World Health Organization (WHO) in collaboration with the US Alcohol, Drug Abuse, and Mental Health Administration (ADAMHA) and translated into German language on behalf of WHO [27]. As a key objective of this study, the Composite International Diagnostic Interview for Oncology (CIDI-O) was adapted for oncology patients based on the Composite International Diagnostic Interview (M-CIDI) in the DIA-X version. The CIDI-O is a standardized computer-based interview which enables the diagnosis of mental disorders according to the ICD-10 and DSM-IV in a reliable, valid and efficient manner. The following mental disorders are included: organic, including symptomatic, mental disorders (F00-F09), mental and behavioral disorders due to psychoactive substance use (F10-F19), mood (affective) disorders (F30-F39), neurotic, stress-related and somatoform disorders (F40-F48) and behavioural syndromes associated with physiological disturbances and physical factors (F50-F59).

To compare the specific psychometric properties (e.g. sensitivity and specificity) of screening measure for anxiety and depression in cancer populations, the HADS as well as the PHQ-9 and the GAD-7 are used.

#### Patient health questionnaire (PHQ-9)

Depression is measured through use of the PHQ-9 German version, the depression module of the Patient Health Questionnaire [28,32]. The PHQ-9 is based on the DSM-IV diagnostic criteria for major depressive disorder and has excellent reliability, as well as criterion, construct, factorial, and procedural validity. The 9 items assess the frequency of depressive symptoms within the past two weeks. Items are scored on a four-point Likert scale rated from 0 (not at all) to 3 (nearly every day) with a total score ranging from 0 to 27. A cutoff point of >9 is recommended for the screening of any depressive disorder including adjustment disorder with depressed mood with a sensitivity of 87% (95% CI 79–92) and a specificity of 76% (95% CI 72–80) [33]. In addition to the sum score for measuring depression severity, the PHQ-9 offers a categorical algorithm based on modified criteria of ‘major depressive disorder’, according to DSM-IV. A score up to 4 indicates the absence of depression, scores of 5–9 represent mild, scores of 10–14 represent moderate and scores of 15 and higher represent severe depression [33].

#### General anxiety disorder-scale (GAD-7)

The GAD-7 German version [34,35] is used to identify probable cases of generalized anxiety disorder and to assess symptom severity. The GAD-7 is based on the most prominent diagnostic features of the DSM-IV diagnostic criteria for generalized anxiety disorder and has excellent reliability, as well as criterion, construct, factorial, and procedural validity. The 7 items assess the frequency of core symptoms of generalized anxiety disorder within the past two weeks. Items are scored on a four-point Likert scale rated from 0 (not at all) to 3 (nearly every day) with a total score ranging from 0 to 21. A score up to 4 indicates the absence of generalized anxiety disorder, scores of 5–9 represent mild, scores of 10–14 represent moderate and scores of 15 and higher represent severe anxiety symptom levels [35].

#### Hospital anxiety and depression scale – German version (HADS)

The HADS is a validated screening instrument for anxiety and depression in somatically ill patients [36] and excludes symptoms that may arise from somatic aspects of illness (e.g. insomnia, weight loss and fatigue). The measure consists of 14 items on a 4-point Likert scale (range 0–3) comprising an anxiety and depression subscale. For both subscales a total score is calculated (ranging from 0–21). A score of 0–7 is categorized as normal, a score of 8 to 10 is considered to indicate a possible anxiety or depressive disorder, and a score of 11 or above is considered to indicate a probable anxiety or depressive disorder.

#### NCCN distress thermometer

The Distress Thermometer is a valid and reliable measure for screening psychological distress in patients with cancer. Initially developed by the National Comprehensive Cancer Network (NCCN) [37] the German version was adapted by Mehnert et al. [38]. The measure contains a single-item visual analogue scale ranging from 0 (“no distress”) and 10 (“extreme distress”) to quantify global level of distress and a standardized symptom checklist. The checklist consists of 36 potential causes of distress (answered ‘yes’ or ‘no’) that are grouped into five categories (practical problems, family problems, emotional problems, spiritual concerns and physical problems). A score of 5 or higher at the visual analogue scale is recommended as a cutoff score for a clinically significant level of distress.

#### Brief pain inventory (BPI)

Pain is assessed on the basis of the Brief Pain Inventory (BPI) [39]. The BPI is a measure developed to assess pain history, pain intensity, and pain interference with a variety of activities. The BPI is well validated among cancer and chronic disease pain patients. In this study, only pain intensity will be analyzed. Pain intensity during the last week is evaluated on a scale ranging from 0 (‘no pain’) to 10 (‘worst pain imaginable’).

#### European organization for research and treatment of cancer quality of life questionnaire (EORTC QLQ – C30)

The EORTC Quality of Life Core Questionnaire EORTC-QLQ-C30 [40] is used to measure cancer-related QOL. The measure incorporates five functional scales (physical, role, cognitive, emotional, social), three symptom scales (pain, fatigue, nausea/vomiting), a global health and quality-of-life scale, and several single items for the assessment of additional symptoms commonly reported by cancer patients (e.g. appetite loss, sleep disturbance) as well as the perceived financial impact of the disease and treatment. The EORTC QLQ – C30 consists of 30 items that are scored on 4-point Likert scales, ranging from 1 (“not at all”) to 4 (“very much”). Two items in the global health and quality-of-life sub-scale are scored on a 7-point linear analogue scale. All functional scales and individual item scores are transformed to a 0–100 scale. Higher scores in the five functional scales and global health status scale represent better functioning, whereas higher scores in symptom scales reflect a greater extent of symptom distress.

#### Illness-specific social support scale short version-8 (ISSS-8)

The Illness-specific Social Support Scale (ISSS) was originally developed by Revenson and Schiaffino [41], and has been adapted to the German language by Ramm and Hasenbring [42]. The newly developed 8-item validated German version [43] of the ISSS measures positive support (4 items) and detrimental interaction (4 items). The two scales ‘positive support’ and ‘detrimental interaction’ show internal consistencies with Cronbach’s alpha = .88 and .68. Items are scored on a 5-point Likert scale ranging from 0 (‘never‘) to 4 (‘always‘).

#### The shared decision making questionnaire (SDM-Q-9)

The 9-item Shared Decision Making Questionnaire (SDM-Q-9) [44] is a brief questionnaire developed to assess the extent of patients’ participation in shared decision making in the medical encounter. The present revised version of the measure consists of nine items developed on the basis of the nine process elements characterizing shared decision making, ranging from the disclosure that a decision needs to be made to a shared decision and arrangements of follow up. In the study version, items are scored on a 5-point Likert scale, ranging from 0 (“strongly disagree”) to 4 (“strongly agree”). Therefore, the SDM-Q-9 score can range from 0 to 36; it is transformed to a 0–100 scale with higher values indicating a higher extent of patients’ participation in shared decision making.

#### Control preference scale (CPS)

The Control preference scale (CPS) [45] encompasses whether decision-making is controlled by the doctor, the patient, or both. It contains five statements on the extent of patients’ preferred participation in decision-making, ranging from an autonomous treatment decision to complete physician responsibility for the decision. Within the questionnaire, a rank order of five statements on the preferred level of participation needs to be indicated. Patients can be categorized into three groups of active involvement (A and B), passive role (D and E) and collaborative role (C).

#### Information and supportive care needs and use of psychosocial support

Questions were developed for this study capturing information needs, sources of information and satisfaction with the information obtained as well as needs for psychosocial support. Use of psychosocial support is assessed through questions covering the type of support offers used, satisfaction with the support, basic attitudes toward psychotherapeutic support, medical referrals and recommendations as well as reasons for non-use of psychosocial support.

### Statistical methods

#### Power calculation

The required sample size was calculated based on the literature-based 4-week prevalence rates of mental disorders in different strata of cancer patients determined from an expected prevalence of 30%. A sample size of 2,400 interviews is required to reduce the standard error of the global prevalence estimate to 1%. To reach 2,400 interviews, 3400 patients needed to be screened by use of the PHQ-9. Following the sample size calculation it was determined that each of the five centers had to enroll approximately 720 patients: 40% each in the inpatient acute and outpatient care and 20% in rehabilitation clinics.

#### Sample stratification

We use a proportional stratified random sample based on the nationwide incidence of all cancer diagnoses in Germany [46]. In this form of stratified random selection, each stratum is represented in the sample in the same proportion as in the population. Thus, this form of stratification allows estimates of mental comorbidity for all major tumor entities and individual care settings. Table 2 shows the cancer incidence rates in Germany, which are the basis for the proportional stratified random sample.

**Table 2:** **Annual cancer incidence statistics for Germany (2003–2004)**[46]

**Cancer site**	**Cancer incidence**					
	**Men(*n*= 230500)**		**Women(*n*= 206000)**		**Total(*N*= 436500)**	
	**n**	**%**	**n**	**%**	**n**	**%**
1. prostate	58000	25.4	0	-	58000	13.3
2. breast	0	-	57000	27.8	57000	13.1
3. colon/rectum	37000	16.2	36000	17.5	73000	16.7
4. lung	33000	14.3	13200	6.4	46200	10.6
5. bladder	21435	9.3	7415	3.6	28750	6.6
6. female genital organs	0	-	27560	13.4	27560	6.3
7. hematological malignancies	12650	5.5	11350	5.5	24000	5.5
8. stomach/esophagus	14900	6.5	8850	4.3	23750	5.4
9. kidney/urinary tract	10750	4.7	6500	3.2	17250	4.0
10. malignant melanoma	6500	2.8	8400	4.1	14900	3.4
11. head and neck	*10600*	*4.6*	*3200*	*1.6*	*13800*	*3.2*
12. pancreas	*6300*	*2.7*	*6600*	*3.2*	*12900*	*3.0*
12. thyroid	*1500*	*0.7*	*3500*	*1.7*	*5000*	*1.2*
12. other	*17865*	*5.6*	*16425*	*8.0*	*34390*	*7.7*

#### Statistical analyses

Potential selection processes will be analyzed by comparisons of responders and non-responders on the basis of demographic and clinical data.

Estimates of the prevalence of mental comorbidity in the target populations are established on the basis of observed prevalence rates. Two kinds of estimates will be reported:

1) Raw (unadjusted) proportions as observed in the total sample or in strata, to allow comparisons with other data sources,

2) Estimates resulting from fitting a predictive hierarchal model to the data that takes into account the design settings as fixed effects and cluster structures as random effects, to allow reliable projections of comorbidity frequencies to defined health care catchment areas or to total Germany.

For projections, the estimated prevalence rates will be extrapolated to the target population by using external or study-specific weight factors. The oversampling of patients for the interviews with high depression scores on the PHQ-9 will be taken into account by a corresponding weighting of the individual prevalence rates as it has been carried out in similar epidemiological studies [21,47].

## Discussion

Primary purpose of this epidemiological multi-center study was to detect the 4-weeks-, 12-months-, and lifetime-prevalence rates of comorbid mental disorders according to the ICD/DSM in cancer patients. We aim to enroll a representative sample of patients in terms of tumor entities and cancer care facilities. On the basis of epidemiological data on the prevalence of mental disorders and distress, the needs and demands for the type and extent of psychosocial support offers can be estimated.

This study was methodologically strong relative to many studies previously conducted. Our study used a cancer-incidence-based recruitment strategy for both genders and a multi-methodological approach including a computer-based structured clinical interview for the assessment of mental disorders including adjustment disorder as well as validated questionnaires for the assessment of subjective emotional distress and supportive care needs. Our targeted sample size is larger than those of earlier studies in cancer patients which allow a variety of subgroup analyses stratified by important risk factors such as cancer progress and limited physical functioning.

We use a statistical model that takes different sources of variability into regard in order to allow reliable projections for cancer comorbidities that have to be faced in Germany over the next years.

In summary, our study will provide a large data set offering detailed and valid information about the specific mental comorbidities, problems and emotional distress among cancer survivors with various tumor entities and disease states. The data further will provide information about specific demographic, functional, cancer- and treatment-related risk factors for mental comorbidity and psychosocial distress, specific supportive care needs and use of psychosocial support offers.

Thus, epidemiological data provide an important basis for the implementation of both information and psychosocial support offers in different health care settings. The identification of predictors for psychosocial support needs in cancer patients allow an early and specific assignment and referral of those patients to adequate psychosocial support offers. Medical health care will be improved by the prevention of chronification of mental disorders and the enhancement of compliance, treatment satisfaction, quality of life and communication between the cancer patient and the health care team.

## Competing interests

The authors declare that they have no competing interests.

## Authors’ contribution

All authors collectively drafted the study protocol and approved the final manuscript. AM is its guarantor.

## Pre-publication history

The pre-publication history for this paper can be accessed here:

http://www.biomedcentral.com/1471-244X/12/70/prepub
